# Circulating protein biomarkers of pharmacodynamic activity of sunitinib in patients with metastatic renal cell carcinoma: modulation of VEGF and VEGF-related proteins

**DOI:** 10.1186/1479-5876-5-32

**Published:** 2007-07-02

**Authors:** Samuel E DePrimo, Carlo L Bello, John Smeraglia, Charles M Baum, Dominic Spinella, Brian I Rini, M Dror Michaelson, Robert J Motzer

**Affiliations:** 1 Translational Medicine, Pfizer Global Research and Development, La Jolla, CA, USA; 2 Clinical Pharmacology, Pfizer Global Research and Development, La Jolla, CA, USA; 3 Clinical Oncology, Pfizer Global Research and Development, La Jolla, CA, USA; 4 University of California San Francisco, San Francisco, CA, USA; 5 Massachusetts General Hospital, Boston, MA, USA; 6 Memorial Sloan-Kettering Cancer Center, New York, NY, USA

## Abstract

**Background:**

Sunitinib malate (SUTENT^®^) is an oral, multitargeted tyrosine kinase inhibitor, approved multinationally for the treatment of advanced RCC and of imatinib-resistant or – intolerant GIST. The purpose of this study was to explore potential biomarkers of sunitinib pharmacological activity via serial assessment of plasma levels of four soluble proteins from patients in a phase II study of advanced RCC: VEGF, soluble VEGFR-2 (sVEGFR-2), placenta growth factor (PlGF), and a novel soluble variant of VEGFR-3 (sVEGFR-3).

**Methods:**

Sunitinib was administered at 50 mg/day on a 4/2 schedule (4 weeks on treatment, 2 weeks off treatment) to 63 patients with metastatic RCC after failure of first-line cytokine therapy. Predose plasma samples were collected on days 1 and 28 of each cycle and analyzed via ELISA.

**Results:**

At the end of cycle 1, VEGF and PlGF levels increased >3-fold (relative to baseline) in 24/54 (44%) and 22/55 (40%) cases, respectively (P < 0.001). sVEGFR-2 levels decreased ≥ 30% in 50/55 (91%) cases and ≥ 20% in all cases (P < 0.001) during cycle 1, while sVEGFR-3 levels were decreased ≥ 30% in 48 of 55 cases (87%), and ≥ 20% in all but 2 cases. These levels tended to return to near-baseline after 2 weeks off treatment, indicating that these effects were dependent on drug exposure. Overall, significantly larger changes in VEGF, sVEGFR-2, and sVEGFR-3 levels were observed in patients exhibiting objective tumor response compared with those exhibiting stable disease or disease progression (P < 0.05 for each analyte; analysis not done for PlGF).

**Conclusion:**

Sunitinib treatment in advanced RCC patients leads to modulation of plasma levels of circulating proteins involved in VEGF signaling, including soluble forms of two VEGF receptors. This panel of proteins may be of value as biomarkers of the pharmacological and clinical activity of sunitinib in RCC, and of angiogenic processes in cancer and other diseases.

## Background

Sunitinib malate (SUTENT^®^, SU11248; Pfizer Inc; New York, USA) is an oral multitargeted tyrosine kinase inhibitor with antiangiogenic and antitumor activity in clinical development for a variety of advanced solid malignancies. It is a potent and selective inhibitor of Class III and Class V split kinase domain receptor tyrosine kinases (RTKs), including VEGFR-1, -2, and -3; PDGFR-α and -β; stem cell factor receptor (KIT); Fms-like tyrosine kinase-3 receptor (FLT3); the RTK encoded by the ret proto-oncogene (RET); and the receptor for M-CSF (CSF-1R) [1-8] each of which have been implicated in tumor cell growth and survival either directly via tumor cell signaling, or, indirectly, via tumor-dependent angiogenesis [9-13].

Sunitinib has been studied in two, independent, open-label phase II studies of metastatic renal cell carcinoma (RCC) [14,15], a highly vascularized disease that accounts for more than 30,000 new cases of cancer and more than 12,000 deaths in the United States each year [16]. In both studies, patients received repeated 6-week cycles of treatment, each comprising sunitinib 50 mg/day administered using a 4/2 schedule (4 weeks on treatment, 2 weeks off treatment), and all patients had prior treatment with at least one cytokine-based therapy. In the first study, in which 63 patients were treated with sunitinib, 40% achieved a partial response (PR), as defined by Response Evaluation Criteria in Solid Tumors (RECIST), and 27% demonstrated stable disease (SD) ≥3 months; the median time to tumor progression was 8.7 months [14]. In the second phase II study, in which 106 patients were treated with sunitinib (1 patient was excluded from the efficacy analysis), the overall investigator-assessed objective response rate was 44%; one patient (1%) achieved complete response and 45 patients (43%) a partial response [15]. Based on these findings, sunitinib received accelerated approval in 2006 from the US FDA for the treatment of advanced RCC. In addition, the European Medicines Agency (EMEA) granted conditional approval for the treatment of advanced and/or metastatic RCC after failure of interferon alfa or interleukin-2 therapy.

With the advent of molecularly targeted therapies and the parallel development of comprehensive integrated staging systems for metastatic RCC, the introduction of molecular tumor markers has the potential to considerably improve attempts to individualize patient prognostication and treatment strategies [17]. The purpose of this study was to explore potential biomarkers of sunitinib pharmacological effect and biologic activity via assessment of plasma levels of four soluble proteins, initially identified in sunitinib phase I studies as potential biomarkers. They include VEGF-A, soluble VEGFR-2 (sVEGFR-2), and placenta growth factor (PlGF; a member of the VEGF family and a specific ligand of VEGFR-1 [18]), all of which are components of the angiogenesis system [10,11,19] and which have previously been reported as circulating factors that are modulated in cancer patients treated with sunitinib [14,20]. Another candidate biomarker evaluated in this study is a novel soluble variant of VEGFR-3 (soluble VEGFR-3; sVEGFR-3). VEGFR-3 is thought to primarily function in lymphangiogenesis and may play a role in tumor cell dissemination to the lymphatic system [21,22]. Herein, we describe the biomarker results and explore relationships with drug exposure and clinical response in the first phase II study of patients with metastatic RCC treated with sunitinib.

## Methods

### Patients

Sixty-three patients with metastatic RCC and prior treatment with first-line cytokine-based therapy (IFN-a, IL-2) were enrolled in this phase II study. The primary endpoint of the trial was objective response rate, as summarized above [14]. (For a complete listing of eligibility criteria, please see prior publication.)

The study was approved by the institutional review board at each of the seven participating centers and was performed in accordance with the Declaration of Helsinki and Good Clinical Practice Guidelines.

### Study Design and Treatment

The starting dose of sunitinib was 50 mg/day, administered using the 4/2 schedule. It was self-administered orally once daily without regard to meals. Intrapatient dose escalation in increments of 12.5 mg/day (up to 75 mg/day) was permitted in the absence of treatment-related toxicity. Dose reduction for toxicity was allowed to 37.5 mg/day and then to 25 mg/day, depending on severity of toxicity.

### Assessment of Sunitinib Levels and Biomarkers

Plasma concentrations of sunitinib and its active metabolite, SU12662, were determined on days 1 and 28 of cycles 1 to 4. Plasma concentrations of both were determined predose by a liquid chromatography/mass spectrometry method at BASi (West Lafayette, IN), with a lower limit of detection of 0.1 ng/mL [23].

Predose plasma samples were collected on days 1 and 28 of each cycle for assessment of soluble proteins that may be correlates of angiogenic activity and/or pharmacodynamic inhibition of VEGF receptor-mediated signaling [24-26]. Each of the soluble proteins was analyzed with enzyme-linked immunosorbent assay (ELISA) kits (all manufactured by R&D Systems, Minneapolis, MN). The (VEGF)-A ELISA assay measures the VEGF-A165 and VEGF-A121 isoforms. The PlGF assay primarily measures PlGF-1. sVEGFR-2 was quantified with an ELISA that measures the extracellular (soluble) domain of VEGFR-2 [27]. Similarly, an ELISA component kit that measures the extracellular (soluble) domain of VEGFR-3 was employed. Both the sVEGFR-2 and sVEGFR-3 assays are calibrated against recombinant proteins consisting of the full-length extracellular domain of the respective receptors. No cross-reactivity or interference is detected between the two receptors in the ELISA assays. Though the structural details of sVEGFR-2 and sVEGFR-3 remain to be established, plasma-derived sVEGFR-2 has been reported to be heavily glycosylated and to have a molecular weight of ~90 kDa when de-glycosylated; the size is similar to that or insect-derived recombinant sVEGFR-2, implying that endogenous sVEGFR-2 may be similar in structure to recombinant versions of the VEGFR-2 extraceullar domain [27]. All ELISA assays were run under Good Laboratory Practice conditions, and performance specifications of each ELISA were validated for their intended purpose, as per established guidelines [28].

### Data Analysis

Protein plasma concentration data and correlations with drug levels and response data were analyzed with Microsoft Excel, S-Plus Version 6.2, and Spotfire Decision Site; comparison results from Student's t-test or Wilcoxon rank sum tests with P < 0.05 were considered statistically significant. All P-values reported are results of two-sided tests. In hierarchical clustering analysis, all values were log2-transformed prior to unsupervised two-dimensional clustering (Spotfire Decision Site).

## Results

### Clinical Results and Sunitinib Pharmacokinetics

Sixty-three patients were treated with sunitinib. The median age was 60 years, and 55 patients (87%) had clear-cell histology [14]. The objective response rate, the primary endpoint of the study, was 40% (For a full listing of baseline characteristics and summary of efficacy and safety findings, please see prior publication.)

Patients achieved and maintained steady-state trough plasma concentrations (C_trough_) of sunitinib and its active metabolite throughout the dosing periods for multiple cycles. The median total drug C_trough _(sunitinib and SU12662 combined) at steady state in all patients was 84.3 ng/mL [14], which is greater than the 50 ng/mL shown to inhibit target RTKs in preclinical models [3]. Drug concentrations increased during the on-drug periods and decreased during the off periods, with no accumulation observed, across dosing cycles.

### Plasma Levels of VEGF, sVEGFR-2, and PlGF: Baseline Levels and Changes During Sunitinib Treatment

**Figure 1:**
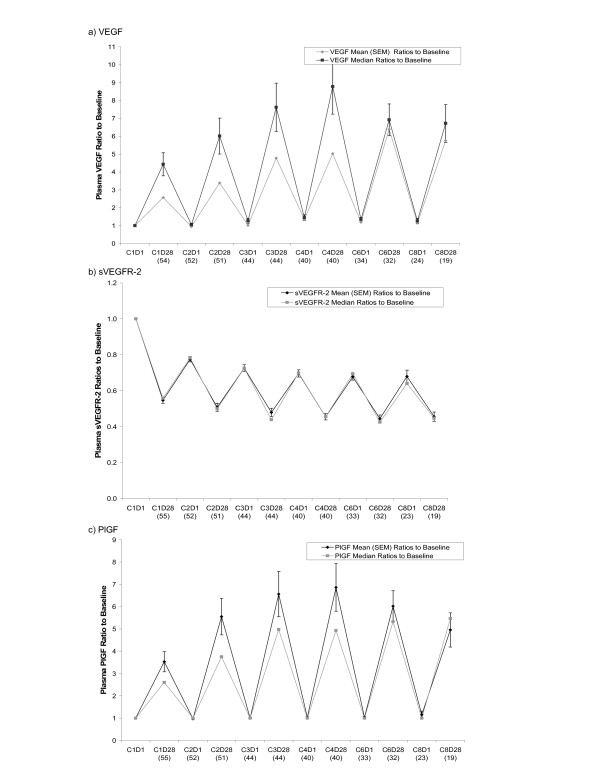
**Mean and median ratios to baseline for VEGF (panel a), sVEGFR-2 (panel b), and PlGF (panel c) at each timepoint in various treatment cycles (SEM = Standard Error of the Mean)**. Mean and median ratios to baseline for VEGF (panel a), sVEGFR-2 (panel b), and PlGF (panel c) at each timepoint in various treatment cycles (SEM = Standard Error of the Mean). Soluble protein plasma level ratios relative to baseline (cycle 1, day 1) are plotted as a series of timepoints, with the baseline values normalized to 1. C(N)D(N) = cycle number, day number (e.g., C1D28 = cycle 1, day 28). The number of values available is listed in parentheses below each time point. The rise and fall of these proteins in accordance with the 4 weeks-on/2 weeks-off sunitinib dosing regimen is indicative of a drug-dependent effect.

Significant changes (p < 0.0001) in the mean plasma levels of VEGF, sVEGFR-2 and PlGF were noted within each dose cycle, as reported in brief elsewhere [14] and as summarized in Table 1 for the first treatment cycle. Baseline (cycle 1, day 1) values for VEGF and sVEGFR-2 were in the assay detection range for all cases; however, in the case of PlGF, only 17 baseline readings (~27%) were measured as above the lower limit of quantitation for the PlGF ELISA assay (at day 28 of cycle 1, 53 of 56 (~95%) cases were in the detectable range). Due to the prevalence of readings below the level of quantitation for PlGF, for all subsequent data analysis such readings were assigned a value equal to the lowest detectable concentration standard (26.2 pg/mL). At the end of the first cycle, VEGF and PlGF levels increased greater than 3-fold relative to baseline (the cycle 1, day 1 timepoint, which is prior to the first sunitinib dose) in 24 of 54 cases (44%) and 22 of 55 cases (40%), respectively. Levels of sVEGFR-2 were decreased by at least 30% in 50 of 55 cases (91%) during the same time frame, and by at least 20% in all cases. For each of these markers, levels tended to return to near-baseline after the 2-week off-treatment period between cycles. This suggests a drug-dependent effect, as illustrated by the patterns of mean and median ratios to baseline as shown for VEGF and sVEGFR-2 (Figure 1a and 1b). Similarly, the results for PlGF clearly indicate a pharmacodynamic effect of sunitinib treatment (Figure 1c), and suggest that PlGF is a biomarker of interest for this agent; however, since most baseline samples (and day 1 samples from subsequent cycles) were below the lower limit of quantification, precise calculation of ratios to baseline was not feasible in most cases.

**Table 1:** Comparison of levels of soluble protein biomarkers (VEGF, sVEGFR-2, and PlGF) at baseline and at the end of the first treatment cycle (day 28 of cycle 1) in RCC patients. Only readings that were in the assay detection range are included in this table.

	**Mean**	**Median**	**Range (pg/mL)**
**VEGF**			
Cycle 1, Day 1 (n = 62)	86.9	67.0	9.7 – 355.5
Cycle 1, Day 28 (n = 55)	278.7	193.3	26.1 – 973.9
**sVEGFR-2**			
Cycle 1, Day 1 (n = 62)	9610.3	9554.5	6390 – 14496.5
Cycle 1, Day 28 (n = 57)	5303.9	5157.0	1948 – 11241.5
PlGF*			
Cycle 1, Day 1 (n = 17)	41.7	35.6	26.7 – 95.9
Cycle 1, Day 28 (n = 53)	111.8	77.9	29.0 – 483.3

### Changes in Plasma Levels of sVEGFR-3

**Figure 2:**
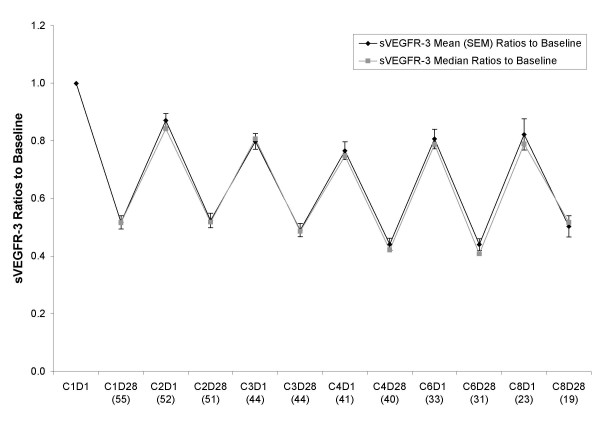
**Mean and median ratios to baseline for sVEGFR-3 at each timepoint in various treatment cycles**. The format follows the same convention as in Figure 1.

In addition to the three angiogenesis-related proteins described above, we also assessed the plasma levels of sVEGFR-3, an apparent circulating variant of VEGFR-3. Assessment of sVEGFR-3 via ELISA was initially selected based on a result of proteomic screening of plasma samples, using an antibody microarray-based multiplex assay [29] in which sVEGFR-3 was identified as a potential pharmacodynamic marker of sunitinib activity [8]. Confirmatory ELISA assessments revealed that significant changes (p < 0.0001) in the mean plasma levels of sVEGFR-3 were noted within each dose cycle (Table 2). Baseline values for sVEGFR-3 were in the assay detection range for all cases; however, at day 28 of the first cycle, sVEGFR-3 levels were reduced to less that the lower limit of quantitation in 12 of 56 cases (21%). Readings below the quantitation limit were frequently observed at day 28 of subsequent cycles as well. For all subsequent data analysis such readings were assigned a value equal to the concentration of the lowest detectable quality control standard (21,200 pg/mL). Levels of sVEGFR-3 were decreased by at least 30% in 48 of 55 cases (87%) during the first cycle, and by at least 20% in all but 2 cases. For each of these markers, levels tended to return to near-baseline after the 2-week off-treatment period between cycles. This suggests a drug-dependent effect, similar to that seen with the other proteins, as illustrated by the patterns of mean and median ratios to baseline for sVEGFR-3 (Figure 2). In the case of VEGF, the magnitude of ratios to baseline at cycle 1, day 28 was higher on average in cases with VEGF baseline values less than the overall median (68.2 pg/mL) compared to cases with higher baseline values (5.7 vs. 3.1; P = 0.039). No significant difference was observed in similar analysis for sVEGFR-2 and sVEGFR-3; this was not done for PlGF due to the detection limitations at baseline for this analyte.

**Table 2:** Comparison of sVEGFR-3 plasma levels at baseline and at the end of the first treatment cycle (day 28 of cycle 1) in RCC patients. Only readings that were in the assay detection range are included in this table.

	**Mean**	**Median**	**Range (pg/mL)**
**sVEGFR-3**			
Cycle 1, Day 1 (n = 62)	71145.2	70700.0	23300 – 129200
Cycle 1, Day 28 (n = 44)	39520.5	37100.0	21500 – 64600

### Correlation Between Biomarker Responses and Drug Exposure

**Figure 3:**
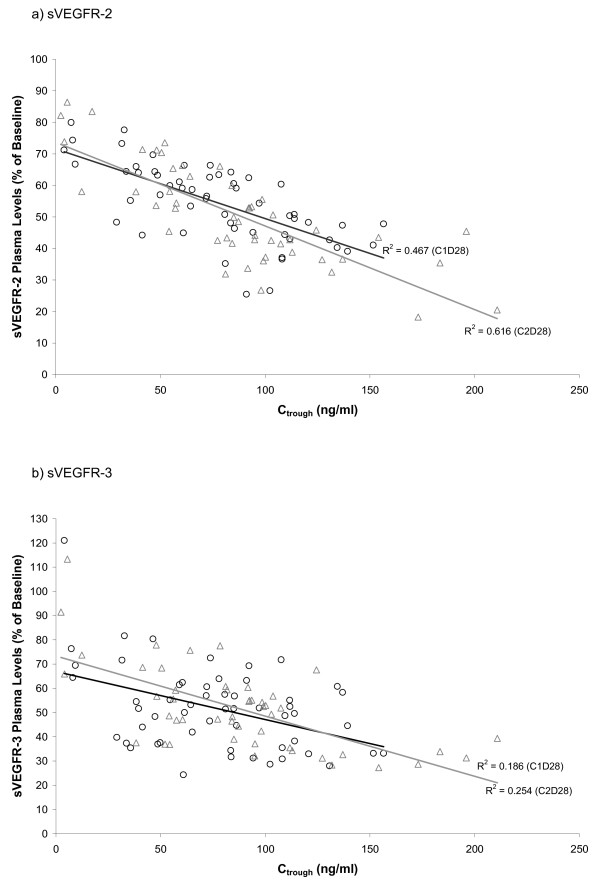
**Correlation between trough drug levels (sunitinib and SU12662, combined) and the intrapatient changes in sVEGFR-2 (panel a) and sVEGFR-3 (panel b) plasma levels during first and second treatment cycles**. (Symbols: О = C1D28; Δ = C2D28)

**Figure 4:**
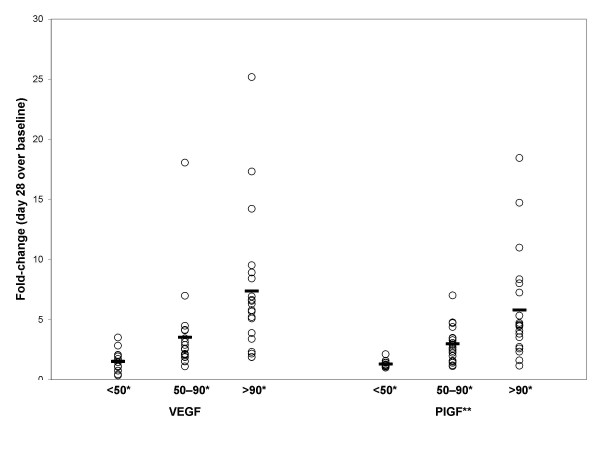
**Fold-changes (ratios to baseline) in mean plasma levels of VEGF and PlGF at different trough levels (x-axis), indicative of a threshold effect**. Fold-change values shown are from cycle 1, day 28 over baseline (cycle 1, day 1). *X-axis categories refer to ranges of combined trough drug levels at day 28 of cycle 1 (in ng/mL); **For PIGF, many of the fold-change values are minimum estimates; values below the lower limit of quantification (LLQ) were assigned value equivalent to the LLQ (26.2 pg/mL)

Exploration of the potential relationships between an indicator of sunitinib exposure (trough drug levels) and the modulation of plasma protein levels was performed. Linear regression analysis revealed a modest correlation between total drug trough drug levels (C_trough _of sunitinib and SU12662, combined) at the end of the first two cycles and the change in mean sVEGFR-2 plasma levels at the end of cycle 1, relative to baseline (Figure 3a). Similar analysis of the change in sVEGFR-3 levels revealed a trend towards greater sVEGFR-3 reduction at higher trough levels (Figure 3b); however, the trend was weaker than that observed for sVEGFR-2. Such a linear trend was not as apparent in similar analysis for VEGF and PlGF modulation (data not shown); however, analyses of the fold-change in mean plasma levels of VEGF and PlGF at different ranges of trough levels indicated a threshold effect with higher mean fold-changes found for trough levels between 50 and 90 ng/mL as compared to those <50 ng/mL, with a further increase at levels >90 ng/mL (Figure 4). This observation suggests a threshold effect for maximal induction of VEGF and PlGF, although there is still considerable overlap in the range of fold-changes across the different trough exposure categories.

### Correlation of Mean Plasma Protein Level Changes with Objective Tumor Response

As summarized above, significant clinical benefit was achieved in this study [14]. To assess potential correlations between the biomarker proteins and objective tumor response, the primary endpoint of the clinical study, a comparison of the biomarker results was performed between the 25 patients with RECIST-defined PR and 32 of the 38 patients with SD or progressive disease (PD). (All cases with baseline and at least one post-treatment timepoint were included). Significantly larger proportional changes in VEGF, sVEGFR-2, and sVEGFR-3 levels were observed in patients exhibiting objective tumor response compared with those exhibiting SD or PD (i.e., 'non-PR') (Table 3); PlGF values were not evaluated in this analysis. The significance was most apparent when only on-treatment ratios (i.e., day 28 of the first 3 cycles) were compared. Analysis in Table 3 is limited to the first 3 treatment cycles, since several patients who did not achieve PR were not treated beyond 3 cycles. It should be noted that while overall, there appeared to be a greater pharmacodynamic effect in the PR group, the patterns of change for these proteins were not discrete enough to allow for definitive response prediction on an individual basis. It is likely that changes in circulating protein levels (detectable here at the first post-treatment sample collection, on day 28) preceded tumor size reductions in at least some cases, since the median time to first observation of partial response was 2.3 months [14]. Baseline plasma levels of these proteins did not differ significantly between the PR and non-PR response groups in this analysis.

**Table 3:** Comparison of changes in levels of soluble protein biomarkers (VEGF, sVEGFR-2, and sVEGFR-3) in patients with partial response (PR) vs. patients that did not achieve partial response (Non-PR). The sample size listed for each group in each case refers to the number of ratios to baseline represented within the different intervals.

	**Mean Fold Change**		**P value**
	**PR (n)**	**Non-PR (n)**	**(t-test; rank sum)**
**VEGF**			
All timepoints (cycles 1–3)	5.22 (123)	2.87 (122)	0.0022; 0.035
All day 28 timepoints (cycles 1–3)	7.91 (74)	3.94 (75)	0.0005; 0.0001
**sVEGFR-2**			
All timepoints (cycles 1–3)	-1.73 (123)	-1.57 (121)	0.0082; 0.001
All day 28 timepoints (cycles 1–3)	-2.12 (74)	-1.79 (75)	0.0003; 0.0003
**sVEGFR-3**			
All timepoints (cycles 1–3)	-1.63 (123)	-1.51 (123)	0.108; 0.104
All day 28 timepoints (cycles 1–3)	-2.17 (74)	-1.89 (76)	0.010; 0.042

n = number of post-treatment:baseline ratios in each group.

**Figure 5:**
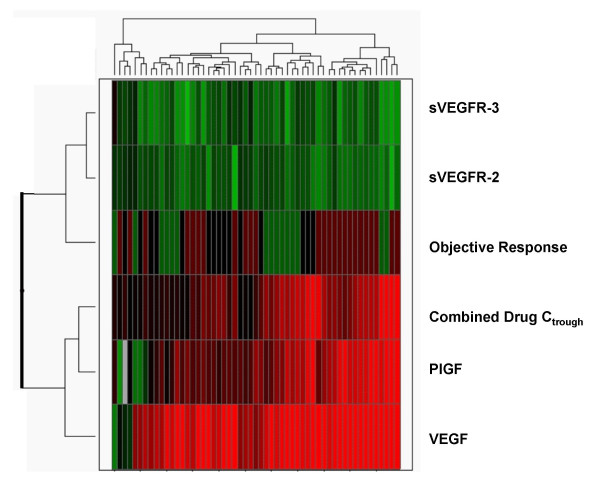
**Hierarchical clustering analysis of soluble protein fold-changes from baseline, drug trough levels, and denotation of best objective clinical responses in each case**. Fold-changes over baseline for VEGF, sVEGFR-2, sVEGFR-3, and PlGF were log2-transformed. C_trough _values were divided by 10 and then log2 transformed. For objective responses, best response of 'PR' was assigned a score of 2, 'SD' was assigned a score of 1, and 'PD' was assigned a score of 0.5, and these assigned scores were also log2-transformed. Red indicates higher values (i.e., positive fold-change or positive response), and green indicates lower values (i.e., reduction from baseline or no response); grey indicates missing values.

For the purpose of illustrating how the patterns of each of these endpoints may be inter-related, hierarchical clustering analysis was performed on the fold-changes for each soluble protein (cycle 1, day 28 over baseline), on C_trough _levels, and on binary scores assigned for best objective tumor response (Figure 5). This 'heat map' depiction indicates that VEGF and PlGF inductions often occur in parallel, though not necessarily to the same extent of magnitude (with the caveat that baseline PlGF levels are often not quantifiable with the ELISA method); similarly, sVEGFR-2 and sVEGFR-3 reductions tend to occur in parallel, but not to the same extent. In this illustration, clustering analysis, using categorical values to represent best objective response outcomes for each patient, suggests that a greater proportion of PR cases are grouped in the range where greater biomarker changes are observed in cycle 1.

## Discussion

This study characterizes circulating levels of the angiogenesis-related proteins VEGF, PlGF, sVEGFR-2, and the novel sVEGFR-3 in patients with metastatic RCC treated with sunitinib, a receptor tyrosine kinase inhibitor with anti-VEGF activity. VEGF and PlGF plasma concentrations increased in many patients after dosing with sunitinib; in contrast, sVEGFR-2 and sVEGFR-3 plasma concentrations were decreased in most patients. Patterns of change for each protein were statistically significant and reproducible over multiple treatment cycles in a cyclical manner, concurrent with the sunitinib dosing schedule and suggesting a dose response. The generally similar effects seen for both VEGF and PlGF are perhaps reflective of sunitinib inhibition of both VEGFR-1 and VEGFR-2, as PlGF is a ligand that specifically binds to VEGFR-1. Changes in each of these four proteins also exhibited some dependence on drug exposure levels as measured by combined trough drug levels, although it is clear that the degrees of change are not strictly correlated with trough drug levels (e.g., note the extent of overlap in VEGF and PlGF ratios between the different trough exposure ranges in Figure 4, despite the significant differences overall). Levels of each protein are restored to near baseline levels by the end of the two-week off-treatment period, during which sunitinib levels are cleared, further indicating that the effects are due to sunitinib activity. Interestingly, the soluble VEGF receptors appear to exhibit a less complete return to baseline levels than do VEGF and PlGF.

Elucidation of the mechanism(s) responsible for VEGF and PlGF induction will require further study, but it is reasonable to speculate that treatment-related increases in tumor hypoxia may be involved, perhaps via increased activity of HIF-1α or other hypoxia-inducible factors. However, the induction may not be completely tumor-dependent, as laboratory studies suggest that VEGF induction following treatment with anti-VEGFR2 monoclonal antibodies can occur in non-tumor-bearing mice [30]. Interestingly, induction of VEGF was not observed in similar experiments where small molecule VEGFR inhibitors were tested (SU5416 and PTK-787; ref. [30]). (It is worth noting that significant induction of circulating VEGF levels has been reported for PTK-787 in a clinical study of colorectal cancer patients [31], and also in another report on results in mouse models [32]; the discrepancy between the mouse study results could be due to differences in experimental design). Also, VEGF induction has been reported in acute myeloid leukemia patients following treatment with sunitinib [26], and induction of VEGF and PlGF has been observed in patients with AML or myelodysplastic syndrome following treatment with the VEGFR inhibitor AG-013736 [33], implying that induction of VEGFR ligands is not limited to patients with solid tumors. The extent of VEGF induction, or lack thereof, can vary widely from case to case despite some correlation with trough drug levels; this implies complex regulation of this factor in response to sunitinib activity and indicates a need for further mechanistic studies. The amount of VEGF at baseline may be one of the contributing factors to the extent of induction.

Sunitinib modulates the plasma levels of two soluble VEGF receptors, sVEGFR-2 and sVEGFR-3, both of which are generally decreased during treatment. Circulating soluble, or shed, receptor fragments have been reported for a large and growing number of receptor tyrosine kinases. These include HER2/erbB-2 [34-36], EGFR [37,38], FGFR1 [39], axl [40], TIE-2 [41], Mer [42], c-Met [43,44], and c-kit [45,46]. These molecules typically are truncated proteins consisting primarily of receptor ectodomains (ECD), and are thought to be primarily shed via proteolytic cleavage at the cell surface. (There is also a soluble form of VEGFR-1, known to be generated via differential splicing of VEGFR-1 mRNA; though this soluble receptor may also be of interest for sunitinib studies, it was not assessed in this study due to lack of an available ELISA assay that would be compatible with the heparinized plasma samples collected here.) Importantly, a number of examples have been reported wherein circulating levels of soluble receptors are modulated in cancer patients by oncology drugs. Such is the case for trastuzumab treatment and soluble Her2/neu ECD in breast cancer [47,48], for soluble EGFR during chemotherapy in breast cancer [49], and for soluble c-kit in response to imatinib treatment in gastrointestinal tumor (GIST) patients [50]. Sunitinib treatment also has shown a longitudinal effect on soluble c-kit levels in GIST as well as other tumor types including RCC [51,52]. To a large extent, the biology of naturally occurring soluble RTK is not well understood, in terms of their genesis, their roles in regulating signaling pathways and ligand bioavailability, or their potential roles in various disease processes.

The strikingly consistent longitudinal changes elicited in sVEGFR-2 and sVEGFR-3 by sunitinib treatment suggests that these changes may be directly reflective of VEGF receptor inhibition, though it is unknown whether this involves changes in receptor synthesis, turnover, proteolytic cleavage, or a combination of mechanisms. Increased receptor internalization triggered by sunitinib binding may not be likely to be a factor, as indeed it is VEGFR-2 activation (autophosphorylation) which is believed to lead to receptor internalization, not its inhibition [53]. Another small molecule VEGFR inhibitor, SU5416, has been shown to not directly affect the cell surface expression of VEGFR-2 [54]. Preliminary experiments evaluating the effect of sunitinib treatment of human umbilical vein endothelial cell (HUVEC) cultured *in vitro *indicate that levels of sVEGFR-2 in cell culture conditioned media are actually moderately increased (~25%) after 72 hours treatment with 10–100 nM sunitinib (data not shown); this suggests that HUVEC cells do not recapitulate the effect of decreased sVEGFR-2 plasma levels observed in patients treated with sunitinib, and the impact of sunitinib on reducing HUVEC cell viability or proliferation rate in culture may influence the observed effect. Further investigation into the processes that underlie the regulation of the soluble receptors and ligands is required, with *in vivo *model approaches perhaps likely to be most informative as they provide an opportunity to measure the effects in the context of full physiological and anatomical microenvironments.

The pharmacodynamic effects on the circulating proteins in this study are generally consistent with preliminary results from similar biomarker analyses in phase II studies of metastatic neuroendocrine tumors (NET) [55] and metastatic breast cancer (mBC) [56] in which sunitinib was administered using the same dose and schedule. For 106 evaluable patients with metastatic NET, VEGF levels were increased more than 3-fold (compared with baseline) in ~50% of patients, and sVEGFR-2 and sVEGFR-3 levels were significantly decreased by greater than 30% in ~60% and 70% of all patients, respectively (P < 0.0001). The reduction in sVEGFR-3 levels was also moderately correlated with tumor response in this study [55]. In the study of mBC, with biomarker data for 62 patients, VEGF levels were increased more than 3-fold relative to baseline in the majority of cases, while sVEGFR-2 levels were decreased by more than 20% in all but 4 cases [56]. Levels of sVEGFR-3 were decreased by more than 30% in 82% of cases during the first cycle, and preliminary evidence of a trend towards greater clinical benefit was observed in patients with a >20% reduction in sVEGFR-3 at the start of the second cycle [56]. Like RCC, metastatic NET and mBC are highly vascular and characterized by high levels of VEGF/VEGFR [57-59], which may account for the similar observations in these analyses.

In summary, these findings suggest that a panel of circulating proteins have utility as biomarkers of pharmacological and clinical activity. Each of these proteins has a known (in the cases of VEGF and PlGF) or presumed (in the cases of the more novel sVEGFR-2 and -3) role in the regulation of angiogenic activity, and the modulation of plasma levels induced by sunitinib treatment is likely to be directly related, at least in part, to inhibition of VEGF signaling via receptor blockade. Assessment of these biomarker variables may help provide a window into biochemical changes triggered by sunitinib and other anti-angiogenic agents. These biomarkers may also provide insights on the pharmacodynamic activity of sunitinib given in different dosing regimens or dosed in combination with other chemotherapeutic agents or targeted therapies, or on the pharmacodynamic activity of other RTK inhibitors. They may also prove useful in non-clinical mechanistic studies of RTK signaling modulators. Further basic laboratory investigations into the structure, biochemical regulation, and molecular physiology of the relatively novel soluble factors VEGFR-2 and VEGFR-3 is also warranted. Given the clear effect on these four proteins related to sunitinib treatment, it is reasonable to speculate that additional factors are modulated during treatment with anti-angiogenic cancer therapeutics; multiplexed proteomic analysis of plasma or serum samples from laboratory and clinical studies is likely to identify additional candidate biomarkers, some of which may have further utility in measuring biologic effects and perhaps predicting treatment outcome.

## Conclusion

Plasma levels of circulating proteins involved in VEGF signaling were modulated in a phase II study of sunitinib in advanced RCC (n = 63), changes for several of which were also correlated with objective tumor response. Changes induced by sunitinib treatment are likely to be directly related, at least in part, to inhibition of VEGF signaling via receptor blockade. This analysis showed that these proteins could be of value as biomarkers of the pharmacological and clinical activity of sunitinib and other anti-angiogenic agents in RCC, and of angiogenic processes in cancer and other diseases.

## Competing interests

Several of the co-authors are employees of Pfizer, as indicated in the author affiliations, and Drs. Motzer, Michaelson and Rini have received research support from Pfizer. In addition, Dr. Rini has received consulting fees from Pfizer, and Dr. Michaelson has received lecture honoraria from Pfizer. Study was supported by Pfizer.

## Authors' contributions

SED, CLB, CMB, BIR, MDM, and RJM contributed to the conception and design of the study

CMB, BIR, MDM, and RJM contributed to the provision of patients and study materials for the study

SED, CLB, JS, BIR, MDM, and RJM contributed to the acquisition of biomarker and related data

SED, CLB, JS, DS contributed to the analysis and interpretation of the biomarker data and correlations

SED was the primary contributor to drafting of the manuscript, which contributions from all authors for revisions

All authors read and approved the final manuscript
